# Comparative Effectiveness of Miswak and Toothbrushing on Dental Plaque and Gingivitis: A Randomized Controlled Trial

**DOI:** 10.3390/healthcare12212150

**Published:** 2024-10-29

**Authors:** Hoda M. Abdellatif, Mamata Hebbal, Eman Alsagob, Abeer Alsaleh, Aljazy Mwena, Mashael Almusaad, Nassreen Aljehani, Shaden Allhidaan, Sharoq Waleed Alreshaidan

**Affiliations:** 1 Public Health Sciences Department, College of Dentistry, Texas A&M University, Dallas, TX 75246, USA; habdellatif@tamu.edu; 2 Department of Preventive Dental Sciences, College of Dentistry, Princess Nourah bint Abdulrahman University, P.O. Box 84428, Riyadh 11671, Saudi Arabia; mihebbal@pnu.edu.sa (M.H.); abeeralsaleh190@gmail.com (A.A.); 437000443@pnu.edu.sa (A.M.); 437000269@pnu.edu.sa (M.A.); nassreenyousef@gmail.com (N.A.); 437000540@pnu.edu.sa (S.A.); shrouqalru@gmail.com (S.W.A.)

**Keywords:** chewing stick, dental deposits, gingival disease, oral health, oral hygiene, *Salvadora persica*

## Abstract

Background/Objectives: The miswak, crafted from the *Salvadora persica* tree, is a traditional teeth-cleaning twig that has served as a natural substitute for toothbrushes and toothpaste across diverse regions for centuries. This study aimed to evaluate and compare the effectiveness of miswak and a conventional toothbrush in reducing dental plaque and gingivitis over a two-week period. Methods: This two-week randomized, single-blind (clinical investigator), two-parallel-arm clinical trial was conducted at Princess Nourah University College of Dentistry (PNUCD). Stratified random sampling identified sixty participants who were then randomly assigned to two study groups: the miswak group and the toothbrushing group. To facilitate data collection, a self-designed form was employed to document participants’ initials, assigned group, the Silness and Loe plaque index, and the Loe and Silness gingival index. The clinical examination, conducted by two blinded and trained examiners using a mouth mirror and explorer under natural light, aimed to assess the specified indices. The collected data were analyzed using IBM SPSS Statistics, Version 22. Results: Following the intervention, the miswak group exhibited no significant change in the plaque scores (*p* = 0.58) compared to the toothbrush group (*p* = 0.007). A notable rise in gingival score was observed within the miswak group (*p* < 0.001), whereas no significant change was noted within the toothbrush group (*p* = 0.52). Conclusions: Over a two-week period, miswak was successful in controlling dental plaque; however, gingival scores were higher, which could be attributed to the aggressive use of miswak. With proper training, the use of miswak has the potential to contribute positively to gingival health, particularly in regions where it is readily accessible and affordable.

## 1. Introduction

Oral health diseases are among the most prevalent non-communicable conditions affecting individuals worldwide. According to the 2022 Global Oral Health Status Report released by the World Health Organization (WHO), oral health issues affect almost 3.5 billion people around the world. These include untreated dental cavities in primary and permanent teeth, severe periodontal disease, and complete tooth loss. A significant 75% of those impacted reside in middle-income countries [1].

Globally, the prevalence of severe periodontal disease is estimated to be 19% among the adult population [1]. A study conducted in Saudi Arabia reported gingivitis to be present in 100% of adults aged between 18 and 40 years [2]. Moreover, dental caries in permanent teeth has a prevalence of 72.1%, while deciduous teeth show a prevalence of 61.7% among children [3]. Remarkably, the majority of these conditions are preventable through simple and cost-effective measures, such as maintaining good oral hygiene practices.

Oral hygiene practices include the activities and behaviors that an individual embraces to uphold the cleanliness and well-being of their teeth and oral cavity. A vital component of oral hygiene is the fundamental practice of regular toothbrushing, which plays a key role in preventing dental caries, gum disease, halitosis, and other oral diseases.

Toothbrushing has a rich and diverse history, with evidence of dental care dating back thousands of years. Between 3500 and 3000 BC, the Babylonians and ancient Egyptians had begun using frayed twigs to clean their teeth. By 1600 BC, the Chinese were also using chewing sticks as primitive toothbrushes [4]. During the Islamic Golden Age between the 8th and 14th centuries, Islamic scholars highlighted the importance of oral hygiene, leading to the widespread use of miswak sticks [5]. Prophet Muhammad had strongly recommended its use, and in 1987, miswak received further endorsement from the World Health Organization [6,7].

Miswak, spelled “miswaak” or “siwak”, is a dental-cleaning twig made from the *Salvadora persica* tree, commonly known as the arak tree. It is used as a natural substitute for toothbrushes in different parts of the world, particularly in the Middle East, Africa, and South Asia. Chewing on or brushing with miswak is beneficial in removing dental plaque, combating bacteria, and refreshing breath. Its natural fibers facilitate the mechanical cleaning of teeth and gums, and its inherent antimicrobial properties, due to the presence of compounds like triethylamine, Salvadorine, flavodine, saponins, sterol, mustard oil, vitamin C, resins and fluoride, contribute to the reduction in bacteria and the prevention of plaque formation [8]. Additionally, it demonstrates antioxidant activity, analgesic effects and antifungal, antiviral, anticariogenic and anti-inflammatory properties [5,9]. These multi-dimensional features underscore the potential oral health benefits associated with the use of miswak.

Research comparing the effectiveness of miswak and traditional toothbrushes is limited, with most studies focusing on parameters such as plaque index, gingival index, and other oral health measures. In a randomized controlled trial involving dental students, it was reported that cleaning teeth with miswak proved more effective in reducing plaque compared to nylon-bristled toothbrushes [10]. Another study indicated that individuals using miswak demonstrated better periodontal health compared to those using traditional toothbrushes [6]. A study conducted in Lucknow found that while miswak was equally effective as toothbrushing for plaque control, it showed lower levels of gingivitis [11]. Additionally, there are studies suggesting that using miswak may be just as effective as traditional toothbrushing in reducing plaque [12].

However, a study concluded that the miswak cannot serve as a complete replacement for a toothbrush but could be utilized as an adjunctive method. Individuals using miswak sticks were found to have higher plaque scores than those using toothbrushes [13]. Given the scarcity of studies and ambiguity in their findings, this study aimed to investigate the potential effects of miswak use on dental plaque accumulation and gingivitis. It seeks to evaluate the effectiveness of miswak in comparison to conventional oral hygiene practices involving a toothbrush over a two-week period in a randomized clinical trial.

## 2. Materials and Methods

### 2.1. Study Design and Setting

**Figure 1:**
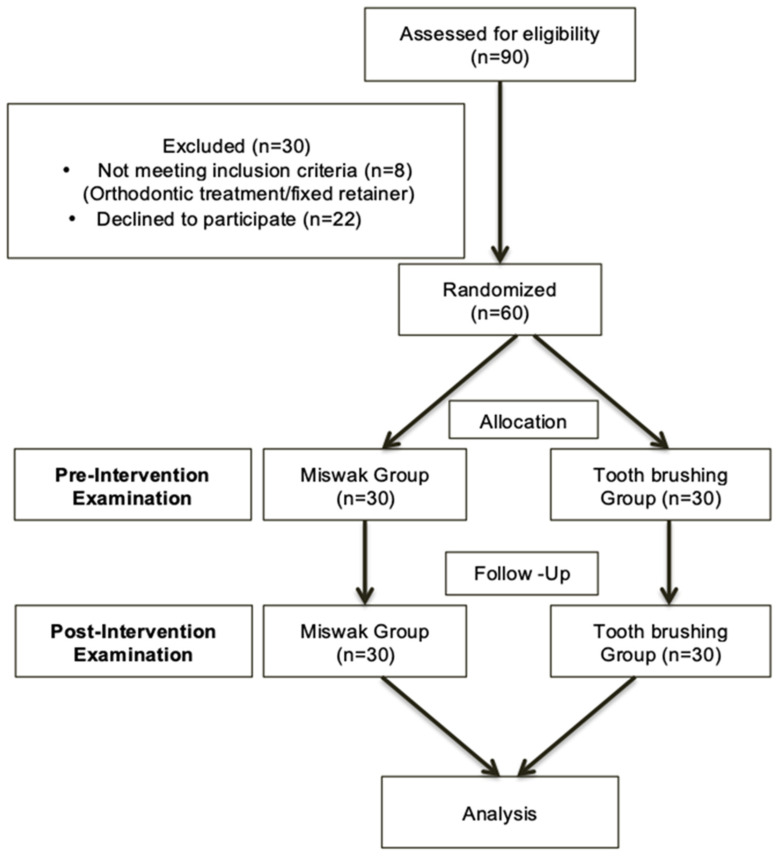
Flowchart of the methodology.

A two-week randomized, single-blind clinical trial with a two-parallel-arm design was carried out at Princess Nourah bint Abdulrahman University College of Dentistry (PNUCD). The study, conducted from January 2023 to February 2023, ensured adherence to ethical standards with prior approval obtained from the institutional review board (IRB) of Princess Nourah bint Abdulrahman University (IRB log number 22-0166). Additionally, the clinical trial was registered with the Thai Clinical Trials Registry (TCTR ID: TCTR20240524005). The methodology is prepared in line with Consolidated Standards of Reporting Trials (CONSORT) guidelines for reporting clinical trials [14]. A flowchart of the methodology is presented in Figure 1.

### 2.2. Sample Size

The sample size was determined using G*Power 3.1 software. In accordance with earlier studies, a standard mean difference of 0.39 was noted in plaque scores, and 0.31 was observed in gingival scores [15]. With a significance level (type I error) set at 5%, a study power of 80% was established, and accounting for a potential loss to follow up of 10%, the determined sample size was 60 participants. This included 30 participants allocated to the miswak group and another 30 participants assigned to the toothbrushing group.

### 2.3. Sample Selection

The study participants were selected from foundation year students, junior dental students, and other students from different colleges who attended the Princess Nourah University (PNU). Since this is a public women’s university, the study participants were exclusively female students. Each student participant provided written informed consent after receiving a thorough explanation about the study. Two examiners conducted an eligibility screening of all students willing to participate in the study using an interview method and clinical examination. Among the eligible students, a total of 60 participants were selected using stratified random sampling based on gingival scores.

Students with adequate oral hygiene (ranging from good to fair), mild to moderate gingival scores, and a minimum of 28 teeth were included in the study. Selected students demonstrated a willingness to abstain from eating or drinking before examinations and were committed to following recommendations and instructions.

Students with any self-reported medical, dental, or psychiatric conditions, abnormalities, or history thereof, as well as those who reported the use of any product or drug that, according to the protocol or investigator’s opinion, might heighten risks associated with trial participation or miswak use or hinder the interpretation of the trial results, were excluded from the study. Additionally, participants were excluded if they had lip, tongue, or other oral piercings or if they utilized fixed or removable orthodontic appliances (like bridges, braces, or dentures). Furthermore, individuals with a history of drugs or treatments reducing salivary flow or currently under recent or ongoing antibiotic coverage were also excluded. It was agreed that any students that used antibiotics or mouth wash or did not comply with the instructions during the study period would be considered as dropouts.

### 2.4. Randomization, Allocation Concealment, and Blinding

Computer-generated random allocation was employed to assign the participants to one of the groups. A list of the 60 participants was prepared and GraphPad software (https://www.graphpad.com/demos/ (accessed on 28 December 2023)) was used for random allocation and to ensure an equal number of participants in each group. A clinical coordinator, not directly involved in the trial, executed this process to ensure impartiality and maintain an even distribution of participants across treatment groups throughout the trial.

To safeguard the randomization sequence, it was concealed from investigators involved in the participant enrollment and assessment. Sequentially numbered, opaque, sealed envelopes (SNOSEs) were utilized, with each envelope containing the treatment assignment for a participant. These envelopes were opened at the time of intervention, revealing the treatment assignments and ensuring that the allocations remained undisclosed until the appropriate trial stage. This approach minimized the risk of biased decision making or inadvertent influence during the participant selection or assessment.

While two investigators engaged in an outcome assessment and the statistician remained blinded to group allocation, study participants were not blinded to their assigned groups.

### 2.5. Interventions

The 60 study participants were randomly assigned to two distinct groups as follows:

- Miswak group (30 participants): participants in this group exclusively used miswak sticks as their plaque control measure.
- Toothbrushing group (30 participants): participants in this group were instructed to rely solely on toothbrushing as their plaque control measure.

A pre-trial training session was conducted for each study group, during which participants were trained and provided instructions, using a model, on the proper use of miswak or toothbrushes according to their assigned group. Participants were provided with miswak sticks measuring 15 cm in length or a soft-bristled toothbrush (19.05 × 1.27 × 1.91 cm) based on their group assignment.

Clear instructions were provided on the proper handling of miswak [6,16]. Participants were taught how to prepare the working end of miswak sticks, and it was emphasized that a new working end should be prepared before cleaning their teeth twice daily. Participants were given the flexibility to adopt either the pen grip (three-finger grip) or the palm grip (five-finger grip). Vertical and horizontal movements were encouraged on both buccal and lingual surfaces, with a crucial stipulation that these motions should consistently be away from the gingival margin.

For the toothbrushing group, participants were advised to brush their teeth twice daily using the provided new toothbrush and a pea-sized amount of fluoridated toothpaste (1450 ppm NaF) following the modified bass technique [17].

Daily reminders were sent to each participant, prompting the use of either miswak or a toothbrush, and they were encouraged to maintain a daily diary.

### 2.6. Data Collection Proforma

A self-designed form was created for the systematic recording of participants’ initials, assigned group, Silness and Loe plaque index and Loe and Silness gingival index [17,18]. Additionally, a self-designed questionnaire comprising six questions was developed to evaluate the participants’ acceptance of using miswak.

### 2.7. Clinical Examination

Two trained and blinded examiners performed clinical examinations under natural light using a mouth mirror and explorer. The examiners underwent training and calibration supervised by a Public Health Dentist to ensure consistency. To gauge inter-examiner reliability, ten randomly selected students underwent examinations for plaque and gingival indices by both examiners, revealing a reliability of 92% for plaque index and 90% for gingival index.

Baseline and two-week follow-up clinical examinations were conducted for all participants at the dental clinic. Silness and Loe plaque index scores, as well as Loe and Silness gingival index scores, were recorded during these examinations [18,19]. Participants who visited a dentist, used antibiotics, or did not adhere to trial instructions were considered dropouts.

Upon the completion of the intervention, a self-designed questionnaire was distributed to assess the acceptance of miswak use. Participants requiring oral prophylaxis underwent the procedure after the study concluded.

### 2.8. Statistical Analysis

The gathered data were entered into a Microsoft Excel spreadsheet and analyzed using IBM SPSS Statistics, Version 22 (Armonk, NY, USA: IBM Corp.). Categorical variables were presented as frequency and percentage, while mean and standard deviation were used for continuous variables. As the data showed a normal distribution, parametric tests were utilized for analysis.

To compare plaque and gingival scores between the two study groups, an independent sample *t*-test was conducted. Within each study group, a paired *t*-test was utilized to compare plaque and gingival scores between before and after the intervention. A significance level of *p* < 0.05 was considered statistically significant.

## 3. Results

Sixty female participants, predominantly from the foundation year (70%), followed by dental (23.3%) and other courses (6.6%) at the College of Health, Princess Nourah bint Abdulrahman University, were engaged in this controlled trial. Their ages ranged between 18 and 21 years.

**Figure 2:**
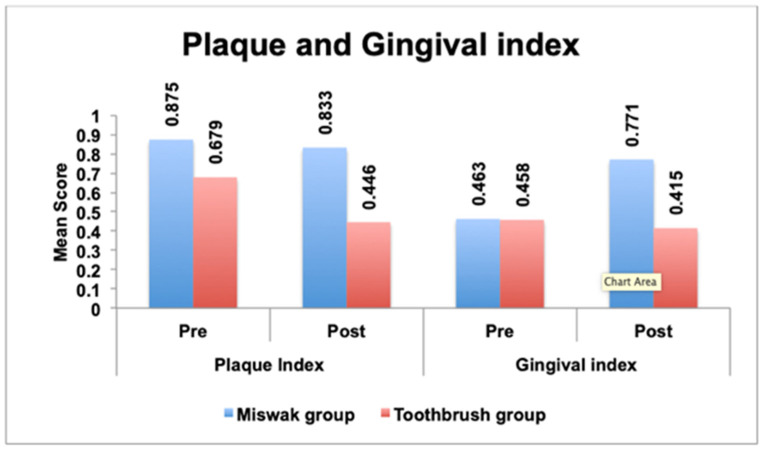
Mean plaque and gingival scores among both groups pre and post intervention.

Prior to the intervention, no significant differences in plaque and gingival scores were noted between the two study groups (*p* = 0.13 and *p* = 0.96, respectively). Following the intervention, the miswak group displayed significantly higher plaque and gingival scores compared to the toothbrushing group (*p* < 0.001). However, there was no significant difference in the change in plaque score between the two study groups (*p* = 0.08). Nevertheless, the change in the gingival score was significantly greater in the miswak group compared to the toothbrush group (*p* = 0.001) (Table 1 and Figure 2).

Upon intragroup comparison, a notable reduction in plaque scores was evident in participants who performed toothbrushing (*p* = 0.007), while no statistically significant change was observed in the miswak group participants (*p* = 0.58). Participants using miswak exhibited an increase in gingival scores post intervention (*p* < 0.001), whereas the decrease in gingival scores in the toothbrushing group did not reach statistical significance (*p* = 0.52) (Table 2, Figure 2).

The majority of the miswak group participants expressed positive responses after its usage. A significant percentage reported its taste as excellent (60%) and found it convenient to use (53.3%). Many participants perceived it as refreshing and effective in eliminating bad breath. A substantial portion (60%) expressed willingness to use it in the future as an adjunct (Table 3).

## 4. Discussion

Toothbrushing remains the foremost and highly effective method for preventing dental caries and gingival diseases. However, despite its efficacy, there exists a considerable global variation in toothbrushing habits. This divergence can be attributed to factors such as insufficient awareness, religious beliefs, cultural customs, economic constraints, and limited access to dental services [20].

This randomized clinical trial was carried out among students enrolled at Princess Nourah University (PNU). During the baseline assessment, both study groups exhibited good plaque scores and mild gingivitis. This observation may be attributed to the exclusive use of toothbrushes among all participants in the study. The predilection for toothbrush usage could potentially be influenced by the relatively younger age group of participants, who might not be as deeply influenced by social and cultural factors emphasizing the importance of miswak use [21].

All 60 participants initially enrolled in the trial completed the study without any dropouts. This high retention rate may be attributed to the relatively short study duration, regular daily reminders sent to participants and their adherence to the protocol, likely facilitated by their shared status as university students who enthusiastically participated in the trial.

Following the intervention, the change in plaque scores was not significantly different between the miswak group and toothbrushing group. The efficacy of miswak in plaque removal is attributed to the mechanical action exerted by its fibers and the antimicrobial effects of compounds present in it, whereas toothpaste has chemical plaque control properties, including stannous fluoride, which plays a role in reducing gingival inflammation, supplementing its mechanical cleansing properties [6,10,22].

Several studies have reported miswak’s effectiveness comparable to toothbrushing in plaque control, while others have found miswak to be superior [6,10,11,21,23,24]. Saudi Arabian subjects showed a significant decrease in *Aggregatibacter actinomycetemcomitans* within the subgingival plaque of miswak users compared to toothbrush users [25]. However, in contrast to the findings of the present study, Patel et al. reported higher plaque scores among miswak users compared to toothbrush users, with no significant difference in gingival scores [13].

The cleaning efficiency of miswak is technique-dependent, requiring good manual dexterity, motivation, knowledge, and attitude on the part of users [16]. When the correct technique is applied, miswak can be as effective as a toothbrush in controlling plaque and managing gingival health [23,26]. Gazi et al. reported a significant decrease in gingivitis and plaque when miswak was used five times daily, whereas plaque scores were higher when used only twice daily [27]. In the current study, participants used miswak twice daily, possibly contributing to the lack of improvement in plaque and gingival status. Miswak’s orientation of bristles along the long axis of teeth, particularly challenging for posterior teeth access, requires more time for cleaning compared to a toothbrush, influencing plaque removal efficiency [28].

The limited duration of the study may have contributed to minimal changes in gingival scores within the toothbrush group. However, in the miswak group, a noteworthy increase in gingival score was observed without a significant change in plaque status. This increase may be attributed to the hard bristles’ inability to reach deep sulcular areas and the potential difficulty of using miswak sticks in posterior segments compared to anterior segments [8,13,16]. Participants in miswak group, accustomed to conventional toothbrushes before the study, may have insufficient experience with miswak. Though adequate care was taken to train participants in the proper technique of using miswak, the participants might have used the miswak aggressively, leading to gingival injury.

Studies conducted in Sudan and India have reported better gingival health among miswak users compared to toothbrush users [11,29]. Nonetheless, a meta-analysis concluded that there was no notable discrepancy in the reduction in plaque and gingivitis between miswak and toothbrush use. However, when miswak was used as an adjunct to toothbrushing, it led to better plaque and gingivitis reduction compared to toothbrushing alone [15]. The severity of plaque accumulation also influences the effectiveness of toothbrushes and chewing sticks, with toothbrushes more effective in heavy plaque deposits, and chewing sticks equally effective in subjects with moderate plaque deposits [30].

As the participants were aware of their participation in the study, it is important to acknowledge the potential impact of the Hawthorne effect on the study outcomes [31]. The participants might have consciously taken measures to enhance their oral hygiene, knowing that they were under observation. Notably, miswak users might have devoted a longer time to teeth cleaning, potentially influencing the outcomes of the study.

The present study had a sufficient timeframe to meet its objectives. However, future research could explore the long-term impacts of miswak on gingival diseases and dental caries. Additionally, exploring the potential use of miswak as an adjunct to toothbrushing warrants further investigation. There is also a scope for designing miswak with improved angulation to enhance accessibility, especially in the posterior segments. As the current study included only female participants, future studies can be conducted including participants from both the gender groups to make the findings more generalizable.

Miswak was well accepted by the study participants who expressed a willingness to continue its usage in the future. Its affordability and ready availability make it a practical choice for urban and rural communities in developing countries [15,32]. While contemporary dental practices predominantly involve toothbrushes and toothpaste, the enduring use of miswak in various communities persists as a traditional and natural approach to oral hygiene. In settings where miswak usage aligns with cultural or religious traditions, individuals may seamlessly incorporate miswak into their daily oral care routines. This cultural acceptance plays a pivotal role in fostering adherence to oral care practices, potentially contributing to enhanced plaque control [5,8,33].

## 5. Conclusions

Over a two-week period, miswak was successful in controlling dental plaque; however, gingival scores were higher, which could be attributed to the aggressive use of miswak. With proper training, guidance, and motivation, the use of miswak has the potential to contribute positively to gingival health.

Given its affordability and cultural acceptance, miswak can be considered a practical oral hygiene tool, especially in low socioeconomic communities. However, it is recommended that miswak be used as an adjunct to conventional toothbrushing rather than a complete substitute. Further long-term studies are needed to explore the impact of miswak on gingival health and its potential role in comprehensive oral hygiene practices.

## Figures and Tables

**Table 1:** Comparison of plaque and gingival scores between study groups at different study intervals.

		Study Groups	N	Mean	SD	Mean Difference	95% CI of the Difference		t	df	*p*-Value
							Lower	Upper			
PI	Pre	Miswak	30	0.875	0.537	0.196	−0.059	0.451	1.54	58	0.13 (NS)
		Toothbrush	30	0.679	0.446						
	Post	Miswak	30	0.833	0.369	0.388	0.221	0.554	4.67	58	<0.001 *
		Toothbrush	30	0.446	0.265						
	Change	Miswak	30	0.042	0.403	−0.192	−0.409	0.025	−1.77	58	0.08 (NS)
		Toothbrush	30	0.233	0.436						
GI	Pre	Miswak	30	0.463	0.328	0.004	−0.165	0.173	0.05	58	0.96 (NS)
		Toothbrush	30	0.458	0.325						
	Post	Miswak	30	0.771	0.377	0.356	0.170	0.541	3.83	58	<0.001 *
		Toothbrush	30	0.415	0.341						
	Change	Miswak	30	−0.308	0.390	−0.351	−0.546	−0.157	−3.62	58	0.001 *
		Toothbrush	30	0.043	0.361						

Notes: Independent sample *t*-test: * *p* < 0.05, statistically significant; *p* > 0.05, non-significant. Abbreviations: PI, plaque index; GI, gingival index; NS, non-significant; CI, confidence interval.

**Table 2:** Comparison of plaque and gingival scores between pre and post intervention in each study group.

	Study Groups		N	Mean	SD	Mean Difference	95% CI of the Difference		t	df	*p*-Value
							Lower	Upper			
PI	Miswak	Pre	30	0.875	0.537	0.042	−0.109	0.192	0.57	29	0.58 (NS)
		Post	30	0.833	0.369						
	Toothbrush	Pre	30	0.679	0.446	0.233	0.070	0.396	2.93	29	0.007 *
		Post	30	0.446	0.265						
GI	Miswak	Pre	30	0.463	0.328	−0.308	−0.454	−0.163	−4.33	29	<0.001 *
		Post	30	0.771	0.377						
	Toothbrush	Pre	30	0.458	0.325	0.043	−0.092	0.178	0.65	29	0.52 (NS)
		Post	30	0.415	0.341						

Notes: Paired *t*-test: * *p* < 0.05, statistically significant; *p* > 0.05, non-significant. Abbreviations: PI, plaque index; GI, gingival index; NS, non-significant; CI, confidence interval.

**Table 3:** Distribution of participants according to acceptance of miswak.

Sl.No	Questions	Options	Frequency	Percentage
1.	How is the taste of miswak?	Excellent (1)	18	60%
		Very good (2)	5	16.67%
		Good (3)	4	13.3%
		Indifferent (4)	1	3.33%
		Bad (4)	1	3.33%
		Very bad (5)	1	3.33%
		Terrible (6)	0	0%
2.	Was the use of miswaak convenient?	Yes (1)	16	53.33%
		No (2)	7	23.33%
		Not sure (3)	7	23.33%
3.	How do you rate using the miswaak help in eliminate bad breath?	Excellent (1)	12	40%
		Very good (2)	9	30%
		Good (3)	2	6.67%
		Indifferent (4)	2	6.67%
		Bad (4)	5	16.67%
		Very bad (5)	0	0%
		Terrible (6)	0	0%
4.	How do you feel after using miswak?	No changes (1)	5	16.67%
		Freshness in the oral cavity (2)	13	43.33%
		Bitterness in the oral cavity (3)	8	26.67%
		Any other (4)	4	13.33%
5.	Are you willing to use it in the future?	Yes (1)	18	60%
		No (2)	4	13.33%
		Maybe (3)	8	26.67%
6.	If yes, Do you want to use it as adjunct to toothbrush?	Yes (1)	15	83.33%
		No (2)	2	11.11%
		Maybe (3)	1	5.55%

## Data Availability

The data used in this study will be available from the corresponding author upon reasonable request.
